# Supplementary material for: Modeling glioblastoma heterogeneity as a dynamic network of cell states
Source: Mol Syst Biol. 2021 Sep 16;17(9):e10105. doi: 10.15252/msb.202010105 (PMC8444284; doi:10.15252/msb.202010105)
Supplement: Supplementary file 5 — Source Data for Figure 3 [file MSB-17-e10105-s001.zip › Figure3A_sourcedata/GSEA_3065/nosigdb_state3.GseaPreranked.1622718426028/GANDAL_ALCOHOLADDICTION_DN_PMID29439242.html]

Details for gene set GANDAL\_ALCOHOLADDICTION\_DN\_PMID29439242[GSEA]

|  || Dataset | state3 |
| Phenotype | NoPhenotypeAvailable |
| Upregulated in class | na\_pos |
| GeneSet | GANDAL\_ALCOHOLADDICTION\_DN\_PMID29439242 |
| Enrichment Score (ES) | 0.54740477 |
| Normalized Enrichment Score (NES) | 1.9246964 |
| Nominal p-value | 0.0 |
| FDR q-value | 0.0017917358 |
| FWER p-Value | 0.015 |
Table: GSEA Results Summary

  

Fig 1: Enrichment plot: GANDAL\_ALCOHOLADDICTION\_DN\_PMID29439242      
 Profile of the Running ES Score & Positions of GeneSet Members on the Rank Ordered List

  

| PROBE | GENE SYMBOL | GENE\_TITLE | RANK IN GENE LIST | RANK METRIC SCORE | RUNNING ES | CORE ENRICHMENT || 1 | PTPRZ1 |  |  | 14 | 0.532 | 0.0728 | Yes |
| 2 | GPM6A |  |  | 37 | 0.460 | 0.1347 | Yes |
| 3 | HNRNPU |  |  | 71 | 0.373 | 0.1830 | Yes |
| 4 | FAT1 |  |  | 95 | 0.350 | 0.2294 | Yes |
| 5 | XRCC2 |  |  | 192 | 0.290 | 0.2589 | Yes |
| 6 | SERPINE2 |  |  | 308 | 0.241 | 0.2794 | Yes |
| 7 | ITGAV |  |  | 322 | 0.238 | 0.3111 | Yes |
| 8 | MSMO1 |  |  | 413 | 0.215 | 0.3309 | Yes |
| 9 | ZNF91 |  |  | 447 | 0.210 | 0.3565 | Yes |
| 10 | ELOVL5 |  |  | 450 | 0.209 | 0.3856 | Yes |
| 11 | NAV2 |  |  | 486 | 0.201 | 0.4096 | Yes |
| 12 | SLC4A4 |  |  | 555 | 0.192 | 0.4286 | Yes |
| 13 | SEMA3E |  |  | 570 | 0.190 | 0.4535 | Yes |
| 14 | CHRNA5 |  |  | 579 | 0.189 | 0.4790 | Yes |
| 15 | MEGF10 |  |  | 762 | 0.162 | 0.4806 | Yes |
| 16 | GOLIM4 |  |  | 892 | 0.149 | 0.4867 | Yes |
| 17 | GRIPAP1 |  |  | 959 | 0.141 | 0.4989 | Yes |
| 18 | CYP51A1 |  |  | 976 | 0.140 | 0.5166 | Yes |
| 19 | AMOT |  |  | 1022 | 0.137 | 0.5306 | Yes |
| 20 | GJA1 |  |  | 1160 | 0.125 | 0.5324 | Yes |
| 21 | ANTXR1 |  |  | 1180 | 0.123 | 0.5474 | Yes |
| 22 | HIBCH |  |  | 1943 | 0.079 | 0.4708 | No |
| 23 | EFEMP1 |  |  | 2265 | 0.066 | 0.4430 | No |
| 24 | IDI1 |  |  | 2285 | 0.065 | 0.4499 | No |
| 25 | SFT2D1 |  |  | 3004 | 0.041 | 0.3731 | No |
| 26 | INSIG1 |  |  | 3136 | 0.038 | 0.3633 | No |
| 27 | TNS3 |  |  | 3372 | 0.031 | 0.3406 | No |
| 28 | POU3F2 |  |  | 3388 | 0.031 | 0.3433 | No |
| 29 | EID2B |  |  | 3607 | 0.025 | 0.3217 | No |
| 30 | SUCLA2 |  |  | 3648 | 0.024 | 0.3205 | No |
| 31 | ARHGAP5 |  |  | 3722 | 0.022 | 0.3152 | No |
| 32 | PLS3 |  |  | 4021 | 0.015 | 0.2830 | No |
| 33 | SHROOM4 |  |  | 4159 | 0.011 | 0.2688 | No |
| 34 | ZNF577 |  |  | 4350 | 0.005 | 0.2476 | No |
| 35 | PIK3C2A |  |  | 4362 | 0.005 | 0.2471 | No |
| 36 | DSTYK |  |  | 4371 | 0.005 | 0.2468 | No |
| 37 | TDP1 |  |  | 4404 | 0.004 | 0.2437 | No |
| 38 | ZNF880 |  |  | 4889 | -0.008 | 0.1892 | No |
| 39 | SOX21 |  |  | 5077 | -0.013 | 0.1694 | No |
| 40 | KCNJ16 |  |  | 5242 | -0.017 | 0.1529 | No |
| 41 | FRZB |  |  | 5464 | -0.023 | 0.1307 | No |
| 42 | MSX1 |  |  | 5833 | -0.034 | 0.0931 | No |
| 43 | ZNF394 |  |  | 6010 | -0.040 | 0.0785 | No |
| 44 | CEP19 |  |  | 6407 | -0.053 | 0.0403 | No |
| 45 | TFG |  |  | 6453 | -0.055 | 0.0428 | No |
| 46 | LRRFIP1 |  |  | 6543 | -0.058 | 0.0407 | No |
| 47 | PPCS |  |  | 6669 | -0.064 | 0.0353 | No |
| 48 | METTL21A |  |  | 6824 | -0.072 | 0.0276 | No |
| 49 | BRD7 |  |  | 7062 | -0.085 | 0.0122 | No |
| 50 | PAPOLA |  |  | 7410 | -0.109 | -0.0124 | No |
| 51 | CHMP5 |  |  | 7487 | -0.116 | -0.0049 | No |
| 52 | SAP30 |  |  | 7564 | -0.123 | 0.0036 | No |
| 53 | APOPT1 |  |  | 8129 | -0.211 | -0.0317 | No |
| 54 | FABP7 |  |  | 8536 | -0.354 | -0.0288 | No |
| 55 | AP1S2 |  |  | 8570 | -0.382 | 0.0208 | No |
Table: GSEA details [plain text format]

  

Fig 2: GANDAL\_ALCOHOLADDICTION\_DN\_PMID29439242: Random ES distribution      
 Gene set null distribution of ES for **GANDAL\_ALCOHOLADDICTION\_DN\_PMID29439242**
